# Annual Meetings of the Pennsylvania Association of Dental Surgeons

**Published:** 1847-03

**Authors:** 


### ARTICLE IX.

*Annual Meeting of the Pennsylvania Association of Dental Surgeons*, held at the Philadelphia Museum, Philadelphia, October 6th, 1846.

Association called to order by the President.

Minutes of last stated meeting read and adopted.

Minutes of special meeting of June 9th read and adopted.

The committee appointed to attend to the publication of Dr. R. Arthur's opening address, beg leave to report that they have attended to the duty assigned them. J. D. WHITE, *Chairman.*

The above report was accepted, and the committee discharged.

Committee on plate and seal reported progress, and was ordered to be continued.

The undersigned committee beg leave to report, that they have attended to the duty assigned them by a resolution, at the special meeting in June last. The essay of Dr. J. D. White was received by them, and published in Stockton's Dental Intelligencer. Mr. J. M. Harris declined having his essay published. The above report is respectfully submitted by your committee.

C. C. WILLIAMS,
E. PARRY, and
F. REINSTEIN.

The above report was accepted, and the committee discharged.

On motion, *Resolved*, That the 19th and 20th articles of the by-laws be reconsidered.

On motion, *Resolved*, That the 20th article of the by-laws be amended, by adding to it the words, *or suspended.*

Then, on motion, *Resolved*, That the association proceed to the election of officers.

On motion, *Resolved*, That the names of the candidates be all placed upon one ballot.

A. R. Johnson and J. M. Harris appointed tellers, and, after the ballotting, reported the election, for one year, of the following officers.

| | |
|---|---|
| G. PLANTOU, | PRESIDENT. |
| ELY PARRY, | 1ST VICE-PRESIDENT. |

STEPHEN T. BEAL, 2D VICE-PRESIDENT.
C. C. WILLIAMS, RECORDING SECRETARY.
R. ARTHUR, CORRESPONDING SECRETARY.
F. A. REINSTEIN, TREASURER,

Then, on motion, *Resolved*, That the association proceed to the election of Examining Committee.

Same tellers, after ballotting, reported the election, for one year, of the following:

J. D. WHITE, *Chairman.*
STEPHEN T. BEAL, ROBERT ARTHUR, and
ELY PARRY, J. M. HARRIS.

Oral communication from J. D. White: subject, *arsenious acid*, and discussed by the members present.

Association adjourned. C. C. WILLIAMS, *Rec. Sec'y.*

---

